# The Influence of Donor and Recipient Complement C3 Polymorphisms on Liver Transplant Outcome

**DOI:** 10.1155/2021/6636456

**Published:** 2021-05-23

**Authors:** Maria Pires, James Underhill, Abdel Douiri, Alberto Quaglia, Wayel Jassem, William Bernal, Nigel Heaton, Phillip Morgan, Richard Thompson, J. Michael Tredger

**Affiliations:** ^1^Institute of Liver Studies, King's College Hospital NHS Foundation Trust, Denmark Hill, London SE5 9RS, UK; ^2^Kings College London, School of Population Health and Environmental Sciences, UK; ^3^National Institute for Health Research Comprehensive Biomedical Research Centre, Guy's and St Thomas' NHS Foundation Trust, London, UK; ^4^King's College London Faculty of Life Sciences and Medicine, UK

## Abstract

Despite early reports of an impact of complement C3 polymorphism on liver transplant patient and graft survival, subsequent evidence has been conflicting. Our aim was to clarify the contributions of donor and recipient C3 genotype, separately and together, on patient and graft outcomes and acute rejection incidence in liver transplant recipients. Eight donor/recipient groups were analyzed according to their genotype and presence or absence of C3 F allele (FFFS, FFSS, FSFF, FSFS, FSSS, SSFF, SSFS, and SSSS) and correlated with clinical outcomes of patient survival, graft survival, and rejection. The further impact of brain death vs. circulatory death during liver donation was also considered. Over a median 5.3 y follow-up of 506 patients with clinical information and matching donor and recipient tissue, five-year patient and graft survival (95% confidence interval) were 90(81-91)% and 77(73-85)%, respectively, and 72(69-94)% were rejection-free. Early disadvantages to patient survival were associated with donor C3 F variant, especially in brain-death donors. Recipient C3 genotype was an independent determinant of graft survival by Cox proportional hazards analysis (hazard ratio 0.26, *P* = 0.04), and the C3 F donor variant was again associated with worse liver graft survival, particularly in brain-death donors. C3 genotype did not independently determine rejection incidence, but a greater proportion of recipient C3 F carriers were rejection-free in the circulatory death, but not the brain-death cohort. Cox proportional hazards analysis revealed significant effects of acute rejection on patient survival (hazard ratio 0.24, *P* = 0.018), of retransplantation on rejection risk (hazard ratio 6.3, *P* = 0.009), and of donor type (circulatory-death vs. brain-death) on rejection incidence (hazard ratio 4.9, *P* = 0.005). We conclude that both donor and recipient complement C3 genotype may influence patient and graft outcomes after liver transplantation but that the type of liver donor is additionally influential, possibly via the inflammatory environment of the transplant.

## 1. Introduction

Complement C3 (C3) is a plasma protein central to the actions of the classical, alternative, and lectin pathways of the complement cascade. An allelic site (rs2230199, C364G) in the C3 gene [1, 2] determines the occurrence of either slow (C3S) (80% of Caucasians) or fast (C3F) (20%) allotypes of C3 (p.Arg102Gly), named on the basis of their electrophoretic mobilities [3–5]. After proteolytic cleavage to C3b, a distinct functional impairment of the binding to soluble complement receptor factor H of the C3F (but not C3S) allotype has been identified [6] which may underlie pathological outcomes.

Evidence associating C3F variants with worse outcomes predominates in immune-mediated diseases [2–4, 7–11] such as predisposition to the development of systemic vasculitis [4], partial lipodystrophy [3], IgA nephropathy [7], and age-related macular degeneration [11]. After transplantation, C3 allotypes may be particularly relevant to both systemic complement activity and graft function. In renal transplantation, C3F allotypes from donor grafts have been associated with significantly improved renal graft function and one-year graft survival [12]. However, this benefit was not confirmed in a subsequent study [13].

Hepatic production of complement C3 dominates that at all other sites [14, 15] but modulating roles for extrahepatic production, particularly in antigen-presenting and responsive cells, are increasingly acknowledged [16–22]. The liver is also immunoprivileged, and the characteristics of organ rejection differ from those of kidneys [23]. Therefore, we rationalized that the outcomes of liver transplantation involving complement C3 pathways might be affected differentially by the allelic variants and particularly by the donor or recipient genotype. Earlier, in 430 cases, Dhillon et al. [24] reported a lack of impact of the presence of the C3F or C3S allele in donor or recipient liver on graft outcome or liver function early after liver transplantation. More recently, Valero-Hervás et al. [25] examined how liver recipient C3 allotype affected one-year liver transplant outcomes in 483 cases independently of donor C3. They showed no influence of recipient C3F genotype on patient survival, a benefit to reduced acute rejection incidence but a negative impact on allograft survival, with increased susceptibility to viral infections [25].

The main objective of this study was to ascertain whether there is any association of both donor and recipient C3 genotype (separately and combined) with patient survival, liver graft survival, and acute rejection in the first five years after liver transplantation. Our preliminary study in 395 cases had shown trends but no significant associations [26]. We, therefore, extended that study to 506 cases to increase power and to investigate additionally the association between C3 polymorphism, cold and warm ischemia times (CIT and WIT, respectively), and postoperative liver function. We also studied two different types of hepatic donors—donors after circulatory death (DCDs) and donor after brain death (DBDs)—to assess whether their dissimilar impact on renal transplant outcome [27, 28] also influenced liver transplantation.

## 2. Patients and Methods

### 2.1. Study Design

Research Ethics Committee (REC) approval was granted for the study of stored material (reference number 10/H0805/13) by the Outer South East London REC within the National Research Ethics Service. The research project (KCH10-025) was approved by the Research and Development Department at King's College Hospital, London, UK. Access to liver donor identity and details was not permissible but links between donor samples and their corresponding recipient(s) were available. Recipient confidentiality was maintained according to Caldecott guidelines by pseudoanonymizing identities to the researcher and separating clinical results and laboratory findings in coded databases. The number of patients followed up was sufficient to detect a 20% change in graft or patient survival with a power of 90% and significance of *P* < 0.05.

#### 2.1.1. Liver Recipients

**Figure 1:**
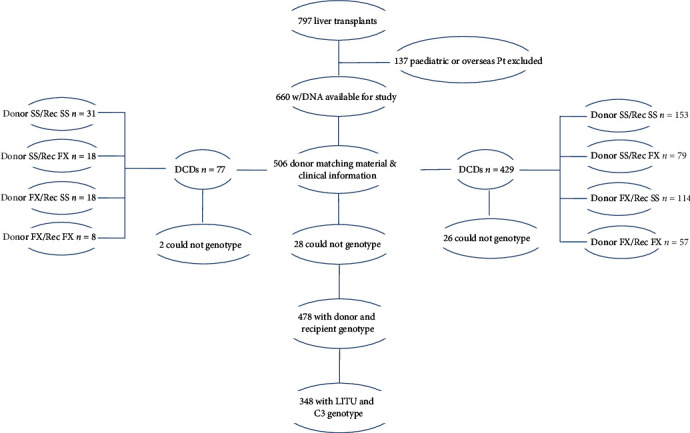
Liver transplant recipients eligible for study inclusion, type of donors, and genotype analysis.

Adult recipients (aged 18-75 years) of one or more liver (only) grafts were selected from the 797 patients who underwent liver transplantation within the King's College Hospital NHS Foundation Trust liver transplant program between January 1, 2005, and December 31, 2010. Overseas patients, children under the age of 18 years, and recipients of living donor grafts were excluded, leaving 660 patients eligible for genotyping (Figure 1). Clinical information on liver recipients was recovered from specialist databases maintained within the Institute of Liver Studies.

Two subgroups of patients were identified whose demographic characteristics were similar to the complete patient cohort. The first subgroup was 498 patients in whom the type of liver donor and corresponding operative details was recorded. The second was 348 patients in whom additional information on surrogates of liver function was available in the first postoperative transplant week.

#### 2.1.2. Liver Donors

No clinical details other than the mode of donor death were accessible for the liver donors because only anonymized stored DNA samples were available. Of the 498 stored donor samples successfully paired with liver recipients, 419 were designated as DBD and 79 as DCD.

### 2.2. Methods

Recipient and donor genomic DNA taken prior to transplantation had been isolated from the leucocyte-enriched buffy-coat interface of EDTA-anticoagulated whole blood. DNA was stored in the Liver Molecular Genetics diagnostic tissue bank at the Institute of Liver Studies, Kings College Hospital at -20°C after performing medium resolution HLA typing. The integrity of the corresponding material had been corroborated by its successful use in NEQAS quality assessment schemes for HLA A, B, DQB, and DRB genotyping. Samples were coded by laboratory number and showed no patient identities. Records of the bank identified 554 matched donor-recipient pairs where sufficient excess donor/recipient DNA was available for genotyping.

#### 2.2.1. C3 Genotyping

Complement C3 C364G polymorphism was detected by amplification refractory mutation system analysis according to the method of Brown et al. [12]. Paired polymerase-chain-reaction (PCR) assays were performed in a DNA Thermal Cycler with 3 *μ*L of genomic DNA. The 3′ base on the sense primers was complementary to the nucleotide substitution associated with the F or S allele. The primers amplified a 278 bp region using the forward primer sequences: 5′AGTTCAAGTCAGAAAAGGTGG3′ (for C3F) and 5′AGTTCAAGTCAGAAAAGGTGC3′ (for C3S) and the reverse common sequence 5′CGTCCGGCCCCACGGGTA3′.

PCR was performed on an automated Applied Biosystem ABi2720 (Foster City, USA) thermocycler. Each PCR run included at least one positive and a negative control PCR product, and each reaction mixture contained control primers, which functioned as internal controls. Amplified products were run through a 2% agarose gel, and the C3 allele was assigned visually. The phenotype was assumed accordingly.

Results were compared in three donors and recipient C3 genotype groups: FF, FS, and SS. There were 9 corresponding C3 donor/recipient combinations: FF/FF, FF/FS, FF/SS, FS/FF, FS/FS, FS/SS, SS/FF, SS/FS, and SS/SS, but no case carried the FF/FF donor-recipient combination. Because of the lower frequency of F alleles, additional comparisons were made where C3F was present (FF or FS, denoted as FX) or absent (SS) for both donor and recipient. This resulted in four corresponding donor/recipient combinations, i.e., C3 FX/FX, FX/SS, SS/FX, and SS/SS.

#### 2.2.2. Clinical Outcomes

Clinical information on recipient outcome to June 30, 2013, was collated in encrypted files and included patient age, sex, race, cause of end-stage liver disease, date of transplant, and number of previous transplants. Key outcomes were patient death and date and graft loss and date (censored for death). Episodes and dates of histologically defined graft rejection were defined as acute steroid responsive or chronic rejection. KCH standard regimens aiming for low calcineurin inhibitor concentrations (“low immunosuppression”) were used in all liver transplant recipients. The same range of maintenance regimens used in all patient groups ciclosporin and prednisolone in transplants prior to 1993 with tacrolimus and prednisolone is progressively superseding that regimen. Corticosteroids were tapered as rapidly and completely as clinically indicated in both cases, and mycophenolate mofetil was used as first-choice supplementary immunosuppression with both regimens if clinically indicated. Azathioprine use was occasional. Identical baseline immunosuppressive therapies were used in recipients of both DBD and DCD donors.

In posttransplant week one, the international normalized ratio of prothrombin time (INR), serum aspartate aminotransferase (AST), serum bilirubin (BIL), and serum lactate (LAC) concentrations were used as indicators of early postoperative liver function. Early graft dysfunction (EGDF) was defined according to Al-Freah et al. [29] on the basis of at least two of the following: AST > 10,000 IU/L, INR > 3.0, and LAC > 3 mmol/L during the first 7 posttransplant days. The criteria of early graft dysfunction of Olthoff et al. [30] were also applied, namely, one of AST > 2000 IU/L during days 1-7, d7 BIL > 10 mg/dl, or d7 INR > 1.6. Primary graft nonfunction was defined as graft failure consistent with a fatal outcome without retransplantation.

#### 2.2.3. Statistical Analysis

Statistical analysis was performed using SSPS software version 22 for Windows (Armonk, NY: IBM Corporation) and by GraphPad Prism version 6.00 for Windows (GraphPad Software, La Jolla, California, USA). Categorical variables were compared by means of Chi-square analysis and continuous variables by means of Kruskal-Wallis and Mann-Whitney *U* tests for nonparametric statistics and by repeat measures analysis of variance (ANOVA). Survival curves among genotype groups were calculated by Kaplan-Meier analysis. Survival results were adjusted for potential confounders using multivariate Cox regression fitted to include the variable donor and recipient C3 genotypes and combinations, mode of donor death, CIT, retransplantation, presence of acute rejection, and selected week one indicators of liver function. For comparisons between C3 genotype subgroups, the SS/SS C3 cohort was selected as a control group. Results were considered significant when the chance of the random occurrence was ≤5% (*P* ≤ 0.05).

## 3. Results

From the 797 liver recipients, 580 were eligible adults with recipient DNA and 506 had matching donor DNA and clinical information (Figure 1). There were 31 retransplants. Of the 31 retransplanted patients, 6 were unavailable to follow-up with 4 having died (2 with recurrent disease/malignancy, 1 from metastatic colorectal carcinoma, and 1 from cardiac failure at retransplant). One further patient was ineligible as a paediatric case, and the sixth moved to Italy and had no further contact with our transplant centre. 11 of the 25 retransplanted patients (44%) experienced acute rejection vs. 125/478 (26.2%) of the total cohort (*P* = 0.06 Fisher's exact test). The majority of acute rejection episodes were mild, and the small number of moderate or severe cases precluded analysis by rejection severity.

In these donor-matched recipients (322 males and 184 females), median age (range) at transplant was 49.9 (17.7–74.2) years. Median follow-up of 5.3 (2.5–8.5) years was to June 30, 2013, during which time 57 patients died (11.9%) and 25 (5.2%) were retransplanted. C3 genotyping was possible in 478 donor/recipient pairs, of which 75 (15.7%) received grafts from donors after circulatory death and 403 (84.3%) from donors after brain death (Figure 1). Week one postoperative liver function data were available in a further subgroup of 348 cases. A histological diagnosis of mild to severe acute rejection was made in 125 (26.2%) cases during standard calcineurin-based immunosuppression, consistent with the literature [31–35]. There were no obvious differences in the indication for transplant between the eight different genotype cohorts.

Table 1 shows a breakdown by C3 genotype of donor, recipient, and donor-recipient combinations for other demographic variables.

### 3.1. Complement C3 Genotype

Of the 478 donor-recipient combinations where successful genotyping was possible, 19 C3 FF donors were transplanted to 0/3/16 C3 FF/FS/SS recipients, respectively. Corresponding figures for the 177 C3 FS donors were 5/56/116 and for the 282 C3 SS donors, 16/82/184 C3 FF/FS/SS recipients, respectively.

Gene frequencies for C3 FF, C3 FS, and C3 SS were 0.04, 0.37, and 0.59 for donors and 0.04, 0.30, and 0.66 for recipients, respectively. Our results show consistency with reported allele frequencies 1 [24, 25] and are in accordance with Hardy-Weinberg equilibrium [36].

### 3.2. Patient and Graft Survival

**Figure 2:**
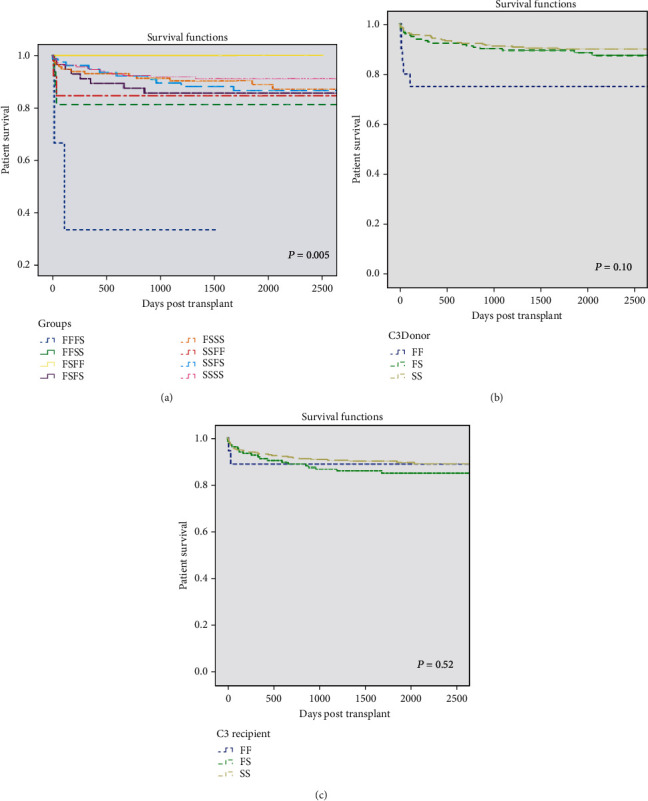
Patient survival by donor/recipient complement C3 genotype. Patient survival numbers at risk and log-rank (*P* values are shown) up to 2500 days. Paired groups (a) and donor and recipient individual groups (b, c) according to C3 genotype and presence of C3F allele (b, c). *P* = 0.005 for genotype groups. (a) Donor's presence or absence of C3F allele, *P* = 0.10; (c) recipient's presence or absence of C3F allele, *P* = 0.52.

Patient survival ranged from 81 to 100% at 2500 days (>6.8years) for all the donor/recipient combinations of the C3 genotype other than FF/FS (Figure 2(a)).

Only 1 of 3 patients with the C3 FF/FS combination survived beyond 110 days.  Figure 2 shows marked early attrition in three of the seven cohorts (C3 FF/FS, FF/SS, and SS/FF), and there was a highly significant difference between patient survival within the different C3 genotypes at 30 d posttransplant (*P* = 0.01 by Mantel-Cox log-rank analysis). These differences persisted to 2500 days (*P* = 0.01) (Table 2).

However, Cox proportional hazards regression analysis showed that C3 genotype was not independently associated with patient survival overall, although there was significantly worse survival in the FS/SS and SS/FS subgroups vs. the reference SS/SS cohort (hazards ratios (HR) 0.21 and 0.28 and *P* = 0.03 and 0.03, respectively) (Table 3).

A separate analysis by the donor or recipient C3 alleles (Figures 2(b) and 2(c)) showed a preponderance of early deaths in both the FF cohorts (80% for donors and 89% for recipients by 90 days vs. ≥95% in both C3 FS and SS subgroups: *P* = 0.002 for donors and 0.42 for recipients). Although the significant survival difference between donor C3 alleles was maintained only to 1-year posttransplant, the donor FF allele showed a persisting survival disadvantage over both FS and SS (Supplementary Table 1). There was no corresponding survival disadvantage over the same time course in donors subdivided for the simple presence of the C3 F allele (FF + FS combined) (Supplementary Table 1, donor FX cohort).

Patient survival to 2500 days in 478 cases stratified according to the presence or absence of the complement C3 F allele showed marginal benefit to the absence of the C3 F allele, which was maintained to 2500 days but did not achieve significance: 81% survival for the C3 SS/SS subgroup vs. 84%, 86%, and 86% in the FX/FX, FX/SS, and SS/FX cohorts (*P* = 0.58 at 2500 days; Supplementary Table 1). There were also no significant differences when survival in the donor and recipient C3 FX and SS groups was compared separately at 2500 days (*P* = 0.33 and 0.28, respectively).

**Figure 3:**
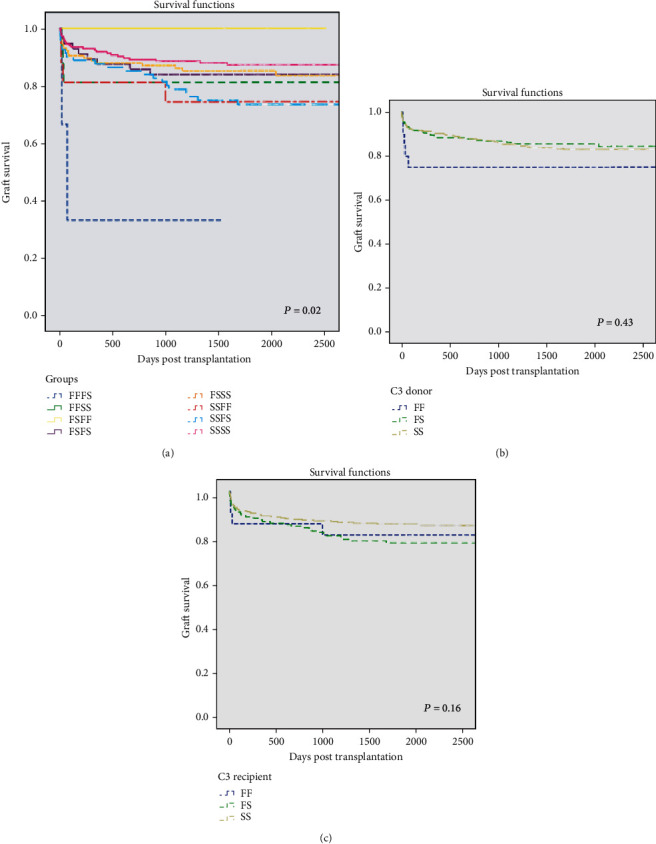
Liver graft survival by donor/recipient complement C3 genotype. Graft survival numbers at risk and log-rank (*P* values are shown) up to 2500 days. Paired groups (a) and donor and recipient individual groups (b, c) according to C3 genotype and presence of C3F allele (b, c). *P* = 0.02 for genotype groups. (a) Donor's presence or absence of C3F allele, *P* = 0.43; (c) recipient's presence or absence of C3F allele, *P* = 0.16.

Liver graft survival was 100% in the 5 C3 FS/FF patients during 1411–2500 days of follow-up. Only 1 of 3 patients with the C3 FF/FS combination retained their graft beyond 110 days. In the remaining six combinations of complement C3 donor/recipient genotype, graft survival ranged from 73 to 84% at 2500 days (>6.8 years) (Figure 3(a)) with marked early graft losses in three cohorts (C3 FF/FS, FF/SS, and SS/FF).

There was a highly significant difference in graft survival between different C3 genotypes at all times between 30 (*P* = 0.03) and 2500 days (*P* = 0.02, Mantel-Cox log-rank analysis) (Table 4). Cox proportional hazards regression analysis showed that C3 recipient genotype was an independent determinant of graft loss (*P* = 0.04 for all groups) and that the recipient F allele significantly impaired survival vs. the SSSS reference cohort (*P* = 0.02 and 0.01 for SS/FF and SS/FS, respectively) (Table 3).

Separate consideration of the impact of donor C3 genotype (Figure 3(b)) showed significantly worse early graft survival in the FF cohort (*P* = 0.02 at 90 days) which was maintained to 6 months but not thereafter (*P* = 0.43 at 2500 days). There were no overall corresponding significant differences for recipient C3 genotype (c), but early attrition in the FF cohort (Figure 3(c)) was reflected by poorer outcome in FF (than FS or SS) recipients of SS grafts up to 2 years (Supplementary Table 2).

Graft survival at 2500 days stratified according to the absence or presence of the C3 F allele was 74-83% in all cohorts (*P* = 0.08; Supplementary Table 2). However, the C3 SS genotype was associated with numerical benefits to long-term graft survival vs. FX in the separate recipient, but not donor cohorts (*P* = 0.06 and 0.90 at 2500 days, respectively).

### 3.3. Acute Allograft Rejection

**Figure 4:**
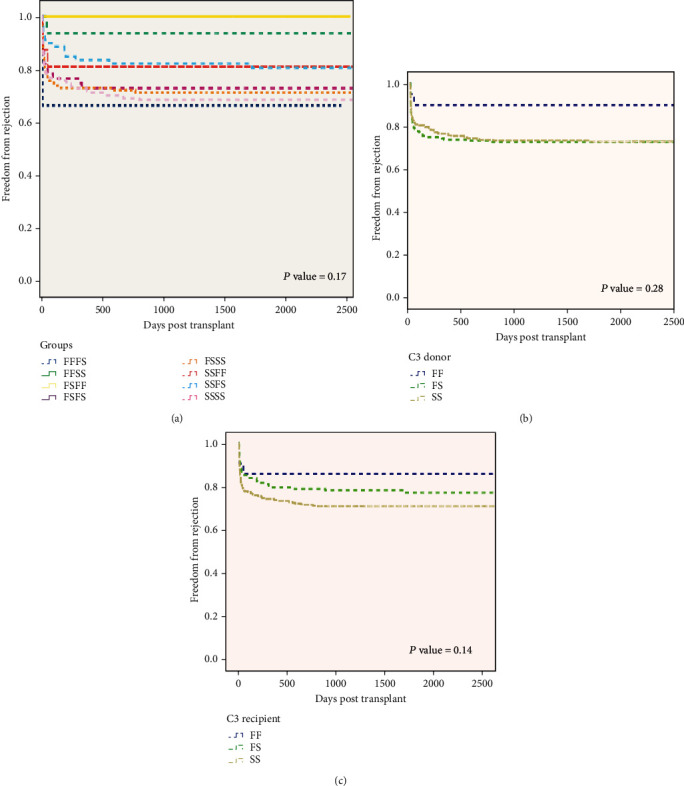
Acute allograph rejection by complement C3 genotype. Freedom from rejection. Numbers at risk and log-rank (*P* values are shown) up to 2500 days. Paired groups (a) and donor and recipient individual groups (b, c) according to C3 genotype and presence of C3F allele (b, c) *P* = 0.17 for genotype groups. (a) Donor's presence or absence of C3F allele, *P* = 0.28; (c) recipient's presence or absence of C3F allele *P* = 0.14.

Kaplan-Meier analysis showed freedom from rejection in all 5 cases within the C3 FS/FF cohort during 1406–2500 days of follow-up (Table 5). The range was 67-94% at 2500 days in the remaining six donor/recipient C3 genotype groups (*P* = 0.17 overall: Mantel-Cox log-rank analysis). The majority of rejection episodes occurred early (<30 days) after transplantation (Figure 4), but again no significant inter-C3 group differences existed (*P* = 0.10). A numerical benefit to freedom from rejection emerged in both the separate donor and recipient C3 FF cohorts, but this failed to reach significance (*P* = 0.28 and 0.14, respectively, at 2500 days). Analysis in relation to the presence or absence of the C3 F allele showed a greater tendency to rejection in the absence of the F allele at all times, but this also failed to reach significance (*P* = 0.12 at 30 days). However, significant differences were evident in the recipient but not the donor cohort (Supplementary Table 3). Cox proportional hazards analysis showed a significant impact of acute rejection on patient survival (HR = 0.280 (0.098-0.805), *P* = 0.02) and of retransplantation on rejection risk (HR 6.3 (1.59–24.9), *P* = 0.01).

### 3.4. Impact of Liver Donation Type on Patient and Graft Survival and Allograft Rejection

Median (interquartile range) cold ischemia time was significantly longer in the DBD than the DCD cohort: 533 (434–648) vs. 420 (335–461) min (*P* < 0.001). CIT was an independent determinant of patient survival in the complete cohort (Cox proportional hazards analysis; *P* = 0.03). Median warm ischemia time (WIT) in the DCD cohort was 16 (13-19) min. Complement C3 allotypes were similarly distributed in DCD and DBD donors, and there were no differences in CIT or WIT between the different genotype cohorts within the respective donor types. The proportion of patients and grafts surviving to 2500 days was higher in DBD than in DCD donors, but these differences were not significant (*P* = 0.43 and 0.69, respectively; Mantel-Cox log-rank test).

Similar results applied at 90 days. The negative impact of donor C3 F achieved higher significance in the DBD than the total cohort (results not shown). Rejection incidence in the two groups was 21.4% vs. 23.4% at 30 days (*P* = 0.79 and similar at 2500 days (*P* = 0.66)). However, there was a lower rejection incidence associated with the recipient C3 F allele in the DCD, but not the DBD cohort (*P* around 0.04 at 30 days and subsequently). Cox proportional hazard analysis showed that results in DBD donors approximated those in the total cohort. Overall, donor type was an independent determinant of acute rejection (HR = 5.303 (1.638-17.17), *P* = 0.01).

### 3.5. Posttransplant Graft Function

Early graft function was investigated in a subgroup of 348 recipients in whom INR, AST, BIL, and LAC results (as surrogates of liver function) were available on days 1-7 posttransplant. Forty-nine (14.1%) of these patients received DCD and 299 (85.9%) DBD grafts. The only test results to differ significantly between DBD and DCD donors were median BIL and INR at d1: 3.19 (0.41–27.0) vs. 2.03 (0.41–12.4) mg/dL, and 1.58 (0.99–5.49) vs. 1.39 (0.97–3.76), respectively. From the complete cohort of grafts, only 3 from DBD donors suffered primary nonfunction: two subsequently died and one was successfully retransplanted. Two recipients of DCD grafts and 9 of DBD grafts suffered initial poor function according to the criteria of Al-Freah et al. [29], and 12 and 56, respectively, by Olthoff criteria [30]. There were no significant differences in the frequency of early dysfunction in DBD vs. DCD donors for either analysis.

**Figure 5:**
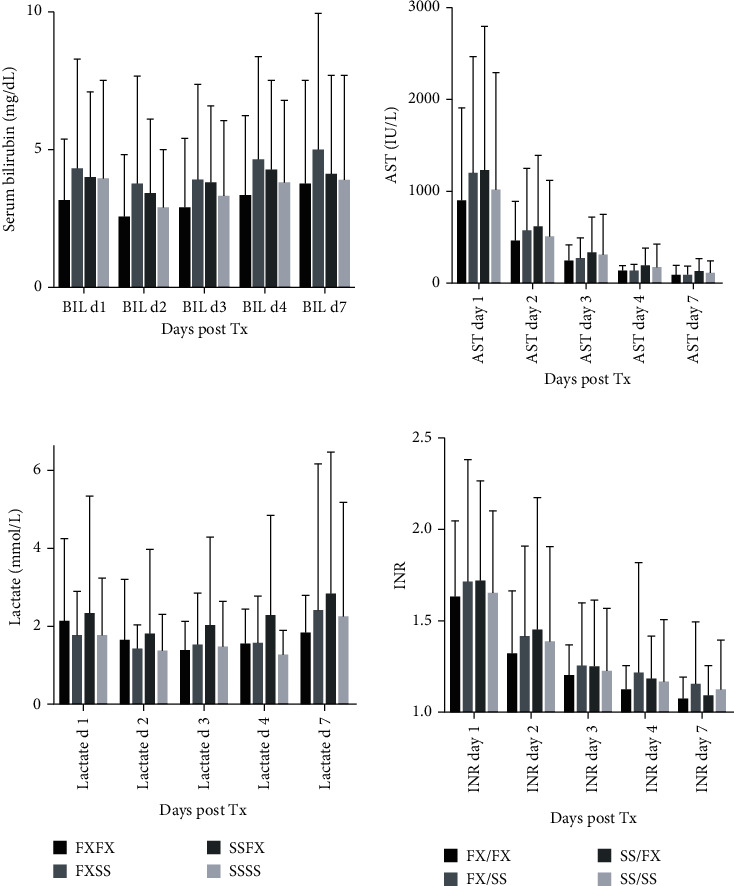
Week 1 liver function by 4 major donor/recipient C3 combinations. Liver function at days 1-7 posttransplant in liver transplant recipients (*n* = 348) classified according to C3 donor/recipient genotype.

The proportions of grafts experiencing early dysfunction also did not differ according to genotype within the DBD and DCD cohorts. Liver function test results in the first week posttransplant stratified according to the presence or absence of the C3 F allele and showed no significant differences by two-way analysis of variance for either donor or recipient genotype. However, a comparable analysis of the four donor/recipient combinations showed that BIL was consistently lowest in the C3 FX/FX and highest in the FX/SS subgroups, while LAC was lowest in the SSSS cohort (Figure 5). These differences between genotypes achieved significance by two-way analysis of variance for BIL (*P* < 0.001) and LAC (*P* = 0.01). Cox proportional hazards analysis showed that a raised LAC on day 4 was associated with worse patient and graft survival and an elevated BIL at day 4 and AST at d7 with impaired graft survival (*P* < 0.03 in every case).

## 4. Discussion

This study has demonstrated that both liver recipient and liver donor complement C3 genotypes differently influence aspects of liver transplant outcome including patient and graft survival, acute rejection episodes, and early liver function. Liver donor status, whether of a DBD or DCD origin, may be additionally influential. Analysis of 478 cases, with unprecedented survival curves for 8 distinct donor/recipient C3 genotype combinations, showed overall differences in both patient and graft survival associated with an early survival disadvantage for the donor FF cohort (versus FS or SS). In contrast, the recipient C3F allele was associated with greater freedom from rejection, significantly so in patients receiving their graft from a donation after circulatory death. Early liver function, including the incidence of week 1 graft dysfunction, appeared largely unassociated with either donor or recipient C3 genotype and differed only marginally in DBD and DCD donors.

Although there were significant differences in patient survival between the eight donor/recipient C3 genotype combinations, and these were sustained over at least 5 years of follow-up, the C3 genotype was not an independent determinant of patient survival by Cox regression analysis. This lack of association, which considers contributions to total systemic C3 of both donor liver and recipient circulatory production, is in agreement with the findings of Dhillon et al. [24] who also considered patient outcome using both donor and recipient genotype. Our separate consideration of the impact of donor or recipient C3 genotype showed a negative impact of donor FF (vs. FS or SS) genotype on patient survival in the first year posttransplant, but not thereafter. Nonhepatic causes, particularly infections, are by far the greatest contributors to mortality in the first posttransplant year [32], consistent with this greater impact of systemic, donor-derived C3F. An impaired survival was observed in the subgroup who donated after brain death but not in the smaller minority of donors after circulatory arrest. In contrast, the recipient C3FF genotype had no significant effect on patient survival, in agreement with Valero-Hervás et al. [25] who studied the impact only of recipient C3 genotype.

Graft survival differed significantly by donor/recipient C3 genotype at all times up to 2500 days posttransplant (Figure 2), both in the total cohort and the subgroup of DBD donors. However, there were no corresponding differences in the smaller cohort of DCD donors. Liver donor C3F genotype was associated with a negative impact on graft survival, with the FF cohort showing worse outcomes than FS or SS donors, irrespective of recipient genotype. Significance was achieved only in DBD donors early (at 60 and 360 days posttransplant) or beyond 5 years. Grafts expressing the greatest C3F load may therefore be more susceptible to local complement-mediated reactions compromising hepatic function and survival, effects which we speculate may be more pronounced in the environment of DBD grafts releasing proinflammatory mediators such as IL-6 [27, 33]. Recipient C3 genotype per se had no significant effect at any time during the posttransplant course, again in agreement with Dhillon et al. [24], but in contrast with Valero-Hervás et al. [25]. The latter authors identified a negative association of recipient C3FF genotype and the C3F allele with graft survival in the first year posttransplant, whereas our data showed only an early attrition in C3FF recipients which was neither significant nor persisted beyond 30 days (Figure 2) and was most evident in the DBD donor subgroup. Notably, our population contained lower numbers of the minority of recipients carrying the C3F allele than in the cases reported by Valero-Hervás et al. [25]. The significant graft survival disadvantage of the recipient C3F variant was especially pronounced in the subgroup of C3FF (vs. FS or SS) recipients of C3SS grafts for up to 2 years. Known immunological effects of the C3 fast allomer may relate to these findings of a negative early impact of both donor and recipient C3F variant on grafts donated from brain-dead (but not DCD) donors. Thus, the fragmented fast C3 allomer is believed to mount weaker alloimmune responses and bind to soluble complement regulator factor H with less affinity than its slow C3S analogue. More potent amplification of the complement alternative pathway is a likely consequence. Early liver graft loss commonly results from surgical complications, thrombosis, primary nonfunction (seen only 3 times in our study), and severe untreatable rejection [34]. While recipient cofactors may modulate these outcomes, the principal causes are graft-related (i.e., of donor origin). Moreover, vascular pathologies are a recognized consequence of cold ischemic injury [35] which was longer in DBD than DCD grafts in this study. Brain death also triggers the autonomic storm and its resultant immune activation, including upregulated complement activation [28], evident prior to kidney donor removal [36], and associated with reduced allograft function in renal transplantation [37]. We speculate that the production of high proinflammatory cytokines present in C3F DBDs contributed to a higher risk of graft loss observed in our study.

Contrasting with the deleterious effect of the C3F allele on graft survival, Valero-Hervás et al. [25] identified a protective effect of the recipient C3F allele on liver graft rejection. Again, we were able to replicate this finding, but only under selective conditions, namely, in the minority subgroup of DCD donors, which was numerically larger in our study population. However, a significant greater freedom from rejection persisted in this cohort across the entire time frame to 2500 days (data not shown). Acute rejection of the liver is commonly an inflammatory process of cellular infiltration of recipient lymphocytes targeted to the donor vascular endothelium and biliary epithelium, sites which are susceptible to ischemic injury. The extended WIT in DCD liver grafts has been associated with necrotic hepatic injury [38], and this may mediate an incompletely understood process of complement activation via the lectin pathway [39]. Responses to the graft may be modulated by the inflammatory environment and abrogated by the suppressive and tolerance functions of nTreg cells promoted by the moderating effect of recipient C3F [40]. Thus, the fast C3 variant exhibits a weaker or less efficient cleavage of C3, lowering the amount of C3a and C3b fragments and delivering weaker stimulatory signals from the engagement of the respective receptors C3aR and CD46 on T effector cells [25, 41]. This, in turn, may cause nTreg cells to differentiate an IL-10-secreting Tr1 population [42] which inhibits the production of the proinflammatory cytokine TNF [40], suppresses or downregulates the induction and proliferation of effector T-cells, and reduces IL-2-dependent IFN*γ* production, so mediating the C3F protective effect of self-tolerance that we observed [42].

## 5. Conclusion

In conclusion, this work extends available knowledge on the protective and detrimental effect of the C3F variant in liver transplantation. By observing that different modes of liver donation, as well as both donor and recipient C3 genotype, may influence the outcome of liver transplantation, previously conflicting data may be rationalized. Unification may be through a process of modulating cytokine profiles via the triggers of the surrounding microenvironment (inflammatory-DBD or necrotic DCD livers) and/or the interaction of distinct C3 alleles with the CD46 receptor on T-cells. Future work should address the deficiencies of this study by recruiting greater numbers of DCD patients and expanding the low numbers within specific donor/recipient combinations, especially for F homozygosity. Finally, direct evidence is required of the altered immune environment related to the triggers of C3 genotype and donor status, perhaps ideally through immunohistological studies on intact tissue. If the molecular signature can be identified, opportunities for therapeutic intervention could arise to direct response from detrimental (effector response) towards protective (tolerance).

## Figures and Tables

**Table 1:** Baseline characteristics of the study population classified by donor and recipient C3 genotype.

	Donors		Recipients		Donor vs. Recipients	
					*P* value	Test
Donor/recipient C3 genotype	SS donor	FF/FS donor	SS recipient	FF/FS recipient		
All cases	282 (59.0%)	196 (41.0%)	316 (66.1%)	162 (33.9%)	<0.001	Chi-square
First transplant (%)	265 (59.3%)	182 (40.7%)	298 (66.6%)	149 (33.4%)	0.02	Chi-square
Repeat transplant (%)	17 (54.8%)	14 (45.2%)	18 (58.1%)	13 (41.9%)	1.0	Fisher's exact
Rejection (% total rejectors)	76 (60.8%)	49 (39.2%)	90 (72.0%)	35 (28.0%)	0.08	Fisher's exact
Rejection free (% by genotype)	206 (73.0%)	147 (75.0%)	226 (71.5%)	127 (78.4%)	0.14	Fisher's exact

Donor/recipient genotype combinations	FX/FX	FX/SS	SS/FX	SS/SS	4 genotypes	Compared
All cases (% total)	64 (13.4%)	132 (27.6%)	98 (20.5%)	184 (38.5%)	—	
Sex (% total)						
Male	39 (12.9%)	82 (27.2%)	63 (20.9%)	118 (39.1%)		
Female	25 (14.2%)	50 (28.4%)	35 (19.9%)	66 (37.5%)	0.96 (M vs. F)	Chi-square
Median recipient age (range) (yr)	54.5 (18.6-70.8)	50.5 (18.0-73.4)	50.2 (18.0-74.2)	53.6 (18.1-72.0)	0.04	Kruskal-Wallis
Rejection (% total)	16 (12.8%)	33 (26.4%)	19 (15.2%)	57 (45.6%)	0.197	Chi-square
Rejection free (% by genotype)	48 (75.0%)	99 (75.0%)	79 (80.6%)	127 (69.0%)		
Donor type						
DBD (*n* = 403)	56 (13.9%)	114 (28.3%)	80 (19.6%)	153 (38.0%)	0.62 vs. DCD	Chi-square
CIT—median (range) (min)	534 (114-1014)	564 (30-1140)	528 (270-906)	540 (215-1200)	0.67	Kruskal-Wallis
DCD (*n* = 75)	8 (10.7%)	18 (24.0%)	18 (24.0%)	31 (41.3%)	0.65 vs. DBD	Chi-square
CIT—median (range) (min)	450 (324-528)	432 (204-558)	420 (174-528)	390 (234-653)	0.87	Kruskal-Wallis
WIT—median (range) (min)	16 (13–29)	16 (9–29)	18 (11–28)	15 (8–24)	0.62	Kruskal-Wallis

**Table 2:** Life tables for patient survival. Patient survival life table up to 2500 days posttransplantation in liver graft donors and recipients classified according to C3 genotype or presence of C3F allele.

C3 subgroup	% survival					
(*n*)	30 d	90 d	180 d	1 yr	5 yr	2500 d
FF/FF (0)	—	—	—	—	—	—
FF/FS (3)	67%	33%	33%	33%	—	—
FF/SS (16)	81%	81%	81%	81%	81%	81%
FS/FF (5)	100%	100%	100%	100%	100%	100%
FS/FS (56)	96%	96%	93%	89%	86%	86%
FS/SS (114)	96%	95%	94%	93%	90%	87%
SS/FF (14)	85%	85%	85%	85%	85%	85%
SS/FS (76)	99%	97%	96%	95%	87%	87%
SS/SS (182)	98%	97%	96%	95%	91%	91%
*P*	0.01	0.01	<0.001	<0.001	0.002	0.01
Donor FF (20)	80%	80%	75%	75%	75%	75%
Donor FS (179)	97%	96%	94%	92%	89%	87%
Donor SS (281)	98%	96%	96%	94%	90%	84%
*P*	0.002	0.002	<0.001	0.003	0.06	0.10
Recipient FF (19)	89%	89%	89%	89%	89%	89%
Recipient FS (135)	97%	96%	93%	91%	85%	85%
Recipient SS (315)	97%	95%	94%	93%	90%	83%
*P*	0.75	0.42	0.59	0.69	0.36	0.52

*P* values were derived by Mantel-Cox log-rank or Wilcoxon rank analysis.

**Table 3:** Cox proportional hazards-derived hazard ratios for patient survival, graft survival, and acute rejection in donor/recipient combinations versus the SS/SS reference cohort.

Donor/recipient	Hazard ratio (95% CI)		
vs. SS/SS	Patient survival	Graft survival	Acute rejection
FF/FS	0.433 (0.027–6.95)	17.0 (0.943–307)	11.3 (0.401–319)
FF/SS	0.002 (0.001–3.17)	0.002 (0.001–1.459)	0.001 (0.001–89.6)
FS/FF	0.890 (0.004–8.93)	0.003 (0.001–18.7)	0.001 (0.001–94.5)
FS/FS	0.430 (0.092–2.01)	1.413 (0.327–6.11)	2.20 (0.752–6.45)
FS/SS	0.205 (0.047–0.890)^∗^	0.919 (0.278–3.04)	1.19 (0.554–2.54)
SS/FF	279 (1.81–43100)^∗^	21.9 (1.69–283)^∗^	1.54 (0.317–7.48)
SS/FS	0.282 (0.089–0.887)^∗^	3.84 (1.32–11.1)^∗^	0.327 (0.076–1.41)

^∗^ *P* < 0.05 Cox regression adjust to donor type, C3 donor, bilirubin day 3, AST day 7, C3 recipient, lactate day 4, C3 recipient, death or alive, lactate day 1, bilirubin day 4, acute rejection, lactate day 3, CIT.

**Table 4:** Life tables for graft survival. Graft survival life table up to 2500 days posttransplantation in liver graft donors and recipients classified according to C3 genotype or presence of C3F allele.

C3 subgroup	% survival						
(*n*)	30 d	90 d	180 d	1 yr	2 yr	5 yr	2500 d
FF/FF (0)	—	—	—	—	—	—	—
FF/FS (3)	67%	33%	33%	33%	33%	—	—
FF/SS (16)	(2)81%	81%	81%	81%	81%	81%	81%
FS/FF (5)	(0)100%	100%	100%	100%	100%	100%	100%
FS/FS (56)	(2)95%	95%	91%	88%	86%	84%	84%
FS/SS (116)	(7)93%	91%	91%	88%	88%	85%	84%
SS/FF (16)	(3)81%	81%	81%	81%	81%	75%	75%
SS/FS (81)	(6)93%	90%	89%	88%	85%	73%	73%
SS/SS (184)	(5)96%	94%	93%	92%	89%	87%	77%
*P*	0.03	0.002	0.005	0.02	0.046	0.009	0.015
Donor FF (20)	80%	75%	75%	75%	75%	75%	75%
Donor FS (182)	94%	92%	91%	88%	88%	86%	85%
Donor SS (290)	94%	92%	92%	90%	88%	83%	77%
*P*	0.15	0.02	0.04	0.08	0.19	0.38	0.43
Recipient FF (21)	86%	86%	86%	86%	86%	81%	81%
Recipient FS (143)	93%	91%	89%	87%	85%	77%	77%
Recipient SS (324)	94%	92%	91%	89%	88%	86%	79%
*P*	0.12	0.57	0.51	0.67	0.71	0.13	0.16

*P* values were derived by Mantel-Cox log-rank or Wilcoxon rank analysis.

**Table 5:** Life tables for freedom from acute rejection. Acute rejection life table up to 2500 days posttransplantation in liver graft donors and recipients classified according to C3 genotype or presence of C3F allele.

C3 subgroup	% rejection-free					
(*n*)	30 d	90 d	180 d	1 yr	5 yr	2500 d
FF/FF (0)	—	—	—	—	—	—
FF/FS (3)	67%	67%	67%	67%	67%	67%
FF/SS (16)	94%	94%	94%	94%	94%	94%
FS/FF (5)	100%	100%	100%	100%	100%	100%
FS/FS (56)	80%	77%	77%	73%	73%	73%
FS/SS (116)	78%	75%	73%	73%	72%	72%
SS/FF (16)	88%	81%	81%	81%	81%	81%
SS/FS (81)	91%	89%	86%	84%	81%	81%
SS/SS (184)	78%	77%	76%	72%	69%	69%
*P*	0.10	0.15	0.23	0.22	0.17	0.17
Donor FF (20)	90%	90%	90%	90%	90%	90%
Donor FS (182)	80%	77%	75%	74%	73%	73%
Donor SS (290)	82%	81%	79%	76%	73%	73%
*P*	0.27	0.41	0.28	0.32	0.28	0.28
Recipient FF (21)	90%	86%	86%	86%	86%	86%
Recipient FS (143)	87%	84%	82%	80%	77%	77%
Recipient SS (324)	79%	77%	76%	74%	71%	71%
*P* value	0.18	0.14	0.19	0.21	0.14	0.14

*P* values were derived by Mantel-Cox log-rank or Wilcoxon rank analysis.

## Data Availability

Supplementary data used to support the findings of this study are included and submitted with the article.

## Supplementary Materials

Supplementary 1 Appendix A: Supplementary Table 1: life tables for patient survival up to 2500 days posttransplantation in liver graft donors and recipients classified according to the presence of the C3 F allele.

Supplementary 2 Appendix B: Supplementary Table 2: graft survival up to 2500 days posttransplantation in liver graft donors and recipients classified according to the presence of C3 F allele.

Supplementary 3 Appendix C: Supplementary Table 3: acute rejection up to 2500 days posttransplantation in liver graft donors and recipients classified according to the presence of C3 F allele.
