# Supplementary material for: The Influence of Donor and Recipient Complement C3 Polymorphisms on Liver Transplant Outcome
Source: Int J Hepatol. 2021 May 23;2021:6636456. doi: 10.1155/2021/6636456 (PMC8168477; doi:10.1155/2021/6636456)
Supplement: Supplementary 1 — Appendix A: Supplementary Table 1: life tables for patient survival up to 2500 days posttransplantation in liver graft donors and recipients classified according to the presence of the C3 F allele. [file 6636456.f1.docx]

## Supplementary Materials

**Appendix A**

**Supplementary Table 1: Life tables for patient survival up to 2500 days post-transplantation in liver graft donors and recipients classified according to presence of the C3 F allele**

| % Survival | 30 d | 90 d | 180 d | 1 yr | 5 yr | 2500 d |
| --- | --- | --- | --- | --- | --- | --- |
| FX/FX (64) | 95% | 95% | 91% | 88% | 84% | 84% |
| FX/SS (130) | 95% | 93% | 92% | 92% | 89% | 86% |
| SS/FX (90) | 97% | 96% | 94% | 93% | 86% | 86% |
| SS/SS (182) | 98% | 97% | 96% | 95% | 91% | 81% |
| *P* | 0.47 | 0.57 | 0.33 | 0.31 | 0.43 | 0.58 |
| Donor FX (195) | 95% | 94% | 92% | 90% | 88% | 86% |
| Donor SS (286) | 98% | 96% | 96% | 94% | 90% | 84% |
| *P* | 0.12 | 0.18 | 0.07 | 0.09 | 0.36 | 0.33 |
| Recipient FX (154) | 96% | 95% | 93% | 91% | 85% | 85% |
| Recipient SS (316) | 97% | 95% | 94% | 93% | 90% | 83% |
| *P* | 0.87 | 0.93 | 0.45 | 0.42 | 0.16 | 0.28 |

***P* values were derived by Mantel Cox log rank or Wilcoxon rank analysis**
