# Supplementary material for: The Influence of Donor and Recipient Complement C3 Polymorphisms on Liver Transplant Outcome
Source: Int J Hepatol. 2021 May 23;2021:6636456. doi: 10.1155/2021/6636456 (PMC8168477; doi:10.1155/2021/6636456)
Supplement: Supplementary 2 — Appendix B: Supplementary Table 2: graft survival up to 2500 days posttransplantation in liver graft donors and recipients classified according to the presence of C3 F allele. [file 6636456.f2.docx]

**Appendix B:**

**Supplementary Table 2: Graft survival up to 2500 days post-transplantation in liver graft donors and recipients classified according to presence of C3 F allele**

| % Survival | 30 d | 90 d | 180 d | 1 yr | 2 yr | 5 yr | 2500 d |
| --- | --- | --- | --- | --- | --- | --- | --- |
| FX/FX (64) | 94% | 92% | 89% | 86% | 84% | 83% | 83% |
| FX/SS (132) | 92% | 89% | 89% | 87% | 87% | 85% | 83% |
| SS/FX (97) | 91% | 89% | 88% | 87% | 85% | 74% | 74% |
| SS/SS(184) | 96% | 94% | 93% | 92% | 89% | 87% | 77% |
| *P* | 0.11 | 0.34 | 0.35 | 0.38 | 0.62 | 0.04 | 0.08 |
| Donor FX (197) | 92% | 90% | 89% | 87% | 86% | 84% | 83% |
| Donor SS (295) | 95% | 93% | 92% | 91% | 88% | 83% | 77% |
| *P* | 0.53 | 0.40 | 0.35 | 0.21 | 0.54 | 0.95 | 0.90 |
| Recipient FX (164) | 92% | 90% | 88% | 87% | 85% | 78% | 78% |
| Recipient SS (324) | 94% | 92% | 91% | 89% | 88% | 86% | 79% |
| *P* | 0.16 | 0.50 | 0.29 | 0.38 | 0.41 | 0.04 | 0.06 |

**P values were derived by Mantel Cox log rank or Wilcoxon rank analysis**
