# Supplementary material for: The Influence of Donor and Recipient Complement C3 Polymorphisms on Liver Transplant Outcome
Source: Int J Hepatol. 2021 May 23;2021:6636456. doi: 10.1155/2021/6636456 (PMC8168477; doi:10.1155/2021/6636456)
Supplement: Supplementary 3 — Appendix C: Supplementary Table 3: acute rejection up to 2500 days posttransplantation in liver graft donors and recipients classified according to the presence of C3 F allele. [file 6636456.f3.docx]

**Appendix C:**

**Supplementary Table 3: Acute rejection up to 2500 days post-transplantation in liver graft donors and recipients classified according to presence of C3 F allele**

| Freedom from Rejection | 30 d | 90 d | 180 d | 1 yrs | 5 yr | 2500 d |
| --- | --- | --- | --- | --- | --- | --- |
| FXFX(64) | 81% | 78% | 78% | 75% | 75% | 75% |
| FXSS(132) | 80% | 77% | 76% | 76% | 74% | 74% |
| SSFX(97) | 91% | 88% | 85% | 83% | 81% | 81% |
| SSSS(184) | 78% | 77% | 76% | 72% | 69% | 69% |
| P value | 0.12 | 0.13 | 0.25 | 0.21 | 0.15 | 0.15 |
| Donor FX (197) | 80% | 78% | 77% | 76% | 75% | 75% |
| Donor SS (294) | 83% | 81% | 79% | 76% | 73% | 73% |
| P value | 0.59 | 0.52 | 0.54 | 0.87 | 0.81 | 0.81 |
| Recipient FX (164) | 87% | 84% | 83% | 80% | 78% | 78% |
| Recipient SS (324) | 79% | 77% | 76% | 74% | 71% | 71% |
| P value | 0.07 | 0.05 | 0.07 | 0.09 | 0.06 | 0.06 |

**P values were derived by Mantel Cox log rank or Wilcoxon rank analysis**
